# Metabolic and transcriptional transitions in barley glumes reveal a role as transitory resource buffers during endosperm filling

**DOI:** 10.1093/jxb/eru492

**Published:** 2015-01-22

**Authors:** Stefan Kohl, Julien Hollmann, Alexander Erban, Joachim Kopka, David Riewe, Winfriede Weschke, Hans Weber

**Affiliations:** ^1^Leibniz Institute of Plant Genetics and Crop Plant Research, 06466 Gatersleben, Germany; ^2^Christian-Albrechts-Universität zu Kiel, 24118 Kiel, Germany; ^3^Max-Planck-Institute of Molecular Plant Physiology, 14476 Potsdam-Golm, Germany

**Keywords:** ABA, barley (*Hordeum vulgare* L. cv. Barke), glumes, jasmonic acid, NAC transcription factors, nitrogen (N) remobilization, N transport, seed development, WRKY transcription factors.

## Abstract

During grain filling in barley (*Hordeum vulgare* L. cv. Barke) reserves are remobilized from vegetative organs. Glumes represent the vegetative tissues closest to grains, senesce late, and are involved in the conversion of assimilates. To analyse glume development and metabolism related to grain filling, parallel transcript and metabolite profiling in glumes and endosperm were performed, showing that glume metabolism and development adjusts to changing grain demands, reflected by specific signatures of metabolite and transcript abundances. Before high endosperm sink strength is established by storage product accumulation, glumes form early, intermediary sink organs, shifting then to remobilizing and exporting source organs. Metabolic and transcriptional transitions occur at two phases: first, at the onset of endosperm filling, as a consequence of endosperm sink activity and assimilate depletion in endosperm and vascular tissues; second, at late grain filling, by developmental ageing and senescence. Regulation of and transition between phases are probably governed by specific NAC and WRKY transcription factors, and both abscisic and jasmonic acid, and are accompanied by changed expression of specific nitrogen transporters. Expression and metabolite profiling suggest glume-specific mechanisms of assimilate conversion and translocation. In summary, grain filling and endosperm sink strength coordinate phase changes in glumes via metabolic, hormonal, and transcriptional control. This study provides a comprehensive view of barley glume development and metabolism, and identifies candidate genes and associated pathways, potentially important for breeding improved grain traits.

## Highlight

The development and metabolism of barley glumes is tightly associated with grain filling and filial sink strength, which coordinate developmental phase changes in the glumes via metabolic, hormonal, and transcriptional control.

## Introduction

During seed filling carbon (C) and nitrogen (N) compounds are remobilized from vegetative organs and transported to the seeds. Vegetative organs in barley and wheat are photosynthetically active, providing carbohydrates until late grain filling. By contrast, 60–90% of grain N originates from remobilization out of vegetative organs (Hirel *et al.*, 2007). Among the photosynthetically active tissues, glumes hold an exceptional role, as the organs nearest the grains. In wheat, glumes have a unique cellular and chloroplast distribution associated with their particular metabolism and supporting grain maturation (Waters *et al.*, 1980; Lopes *et al.*, 2006). Up to 30% of photosynthates imported into grains derive from photosynthetic activity of glumes (Grundbacher, 1963).

Most N derived from other source tissues is not directly transported into developing grains, but instead transported through and eventually converted within glumes (Waters *et al.*, 1980; Simpson *et al.*, 1983).

Compared to flag leaves, glumes senesce late and thus could be important for N translocation during later grain filling. Thereby, the capacity of glumes to convert and translocate N during senescence is an important trait to assess in breeding for higher grain protein content.

To fulfil these tasks, glume metabolism must be coordinated with the different phases of grain development. In barley endosperm, cellularization begins around 4 days after pollination (DAP) and is completed 1–2 days later. The pre-storage/cellularization phase, from anthesis to 6 DAP, and the storage phase, starting at 8–10 DAP, are separated by a transition stage characterized by transcriptional reprogramming and a switch into storage mode (Sreenivasulu *et al.*, 2004). Between 8 and 10 DAP, the endosperm starts accumulating storage products, develops high sink strength for sucrose and N, and enters the linear phase of dry matter accumulation between 10 and 20 DAP (Weschke *et al.*, 2000). Physiological maturity is reached around 24 DAP followed by desiccation.

Endosperm phase changes are accompanied by differences in sink strength, which affect metabolism, remobilization, and transport of resources into and from vegetative organs. As would be expected, central metabolism in glumes is coordinated with such changes during grain filling (Lopes *et al.*, 2006). There is a lack of detailed knowledge about this cross-talk at the molecular level, and of metabolic and transcriptional adjustments according to the specific demands of grains. The remobilization of assimilates and reserves during seed filling is highly regulated (Watanabe *et al.*, 2013). The WRKY (contains the WRKY amino acid signature at the N-terminus and zinc-finger structure at the C-terminus) and NAC (*N*AM, *A*TAF1,2, *C*UC) transcription factors are involved in regulating remobilization and senescence (Balazadeh *et al.*, 2010; Breeze *et al.*, 2011; Fischer, 2012). In barley, specific members of the NAC transcription factor gene family are co-regulated with senescence-associated genes in senescing flag leaves (Gregersen and Holm, 2007; Christiansen and Gregersen, 2014). In wheat the NAC transcription factor Gpc-B1 accelerates senescence and increases nutrient remobilization from leaves (Uauy *et al.*, 2006).

Remobilized N from protein degradation has to be transported across membranes by specific transporters (Tegeder and Rentsch, 2010). Members of the amino acid transporter family (ATF) and nitrate/peptide transporter family (NPF) are key components in remobilization, and functionally characterized transporters are expressed in tissue and development specific-manners. AtAAP1 is involved in amino acid uptake into embryos (Hirner *et al.*, 1998; Sanders *et al.*, 2009), AtAAP8 is involved in amino acid uptake into endosperm (Schmidt *et al.*, 2007), and AtPTR5 is preferentially expressed during early seed development (Komarova *et al.*, 2008). Barley HvPTR1 transports peptides from endosperm to growing embryos during germination (West *et al.*, 1998). Transporters involved in amino acid uptake into cells have mainly been characterized (Tegeder, 2012), but recently AtBAT1 (Dündar and Bush, 2009) and AtSIAR1 (Ladwig *et al.*, 2012) have been shown to export amino acids out of cells with apparently opposing functions. While AtBAT1 shows preferential expression in sink tissues (Dündar, 2009), AtSIAR1 expression is associated with source tissues, and *Arabidopsis* mutants have lower contents and disturbed homeostasis of amino acids in siliques (Ladwig *et al.*, 2012).

The aim of this study was to analyse temporal changes of transcript and metabolite abundances in glumes and endosperm during barley grain development. Such parallel profiling allows a correlation of shifts in glume metabolism and remobilization events with distinct phases of grain development. Furthermore, possible signals and transporters involved in coordinating metabolism and N translocation between glumes and endosperm are presented and discussed.

## Material and methods

### Plant growth and harvest

Barley (*Hordeum vulgare* L. cv. Barke) was grown in greenhouses with 16h light/8h dark. Stages of grain development were determined as described previously (Weschke *et al.*, 2000). Glumes and endosperm tissue were collected between 10am and 12pm in 2- or 4-day intervals starting at anthesis (glumes) and 4 DAP (endosperm) until 24 DAP. Endosperm was manually separated from pericarp between 4 and 14 DAP, and whole caryopses were sampled between 16 and 24 DAP.

### Array design

Transcript data from HarvEST assembly 35 (www.harvest.ucr.edu), two RNAseq experiments (Kohl *et al.*, 2012; Thiel *et al.*, 2012*a*), and a full-length cDNA collection (Matsumoto *et al.*, 2011) were assembled to 46 114 unique barley contigs using TGICL pipeline (http:// compbio.dfci.harvard.edu/tgi/) as described previously (Kohl *et al.*, 2012). Sequences were annotated using Blast2go (Gene Ontology terms) (Conesa *et al.*, 2005), Mercator (bincodes) (Thimm *et al.*, 2004), and BLAST (Altschul *et al.*, 1990). Best hits were obtained from BLASTx similarity searches against UniRef90 (www.uniprot.org), TAIR10 (www.arabidopsis.org), *Oryza sativa* (http://rice.plantbiology.msu.edu/, last accessed 31 December 2014), and UniProtKB/Swiss-Prot (www.uniprot.org). Unambiguous 60bp oligomer probes were derived using eArray (Agilent Technologies, Santa Clara, USA) and a part of this probe set was replicated. Microarray design and expression data is available at EMBL-EBI ArrayExpress, accession E-MTAB-3040.

### RNA isolation, labelling, and array hybridization

Glume and endosperm material for three biological replicates was harvested from 0 (only glumes), 4, 8, 10, 14, 18, and 24 (glumes and endosperm) DAP; and total RNA was extracted with a Spectrum™ Plant Total RNA Kit (Sigma Aldrich, Steinheim, Germany). RNA integrity was confirmed using the Bioanalyser system (Agilent Technologies). 100ng RNA was used for cRNA synthesis and Cy3-labelling with a Low Input Quick Amp Labelling Kit (Agilent Technologies). Labelling efficiency, and amount and quality of cRNA, were assured using an ND-1000 Spectrophotometer (NanoDrop Technologies, Wilmington, USA) and Bioanalyser system. 600ng labelled cRNA was used for fragmentation and array loading (Gene Expression Hybridization Kit, Agilent Technologies). Hybridization was done for 17h at 65°C. After washing (Gene Expression Wash Buffer Kit, Agilent Technologies) and drying, arrays were scanned at 5 µm resolution using an Agilent Technologies Scanner G2505C. Resulting images were evaluated (determination of spot intensities, background correction) with Feature Extraction V11.5 (Agilent Technologies).

### Data evaluation

Data evaluation was done with Genespring V12.5 (Agilent Technologies). Values were log_2_ transformed and quantile normalized, before relative expression values were calculated by subtracting the median expression of each probe from the other values of this specific probe (baseline transformation). After removing outliers and transcripts without significant expression at any time point, ANOVA (*P* ≤ 0.005, *FC* ≥ 3) and FDR correction (Benjamini-Hochberg) was performed. These stringent parameters were chosen in order to identify important transcripts without (unnecessarily) expanding the data set.

### Preparation of vascular tissue

Seeds were harvested (eight biological replicates) at 4, 8, 10, 14, 18, and 24 DAP according to Thiel *et al.* (2009). Vascular tissues were micro-dissected from the middle of the grain with 30 µm thickness per section (Supplementary Figure S1).

### UPLC measurements

Extraction and measurement are described in Thiel *et al.* (2009), with the following changes: 10mg of dried material was used; before extraction, 5 µl Norvalin (5mM) were added; 100 µl aliquots of extract were concentrated under vacuum and dissolved in 200 µl water.

### C/N and starch measurements

Total C and N were determined from dried and ground material using a Vario EL Elementar analyser (Elementar Analysensysteme GmbH, Hanau, Germany). Starch was measured as described (Weschke *et al.*, 2000).

### Metabolite profiling by GC-MS

For GC-MS measurements of polar central metabolites, 100mg (glumes) and 10mg (endosperm) fresh material from six biological replicates was collected in 2-day steps (0–24 DAP) for glumes and at 4, 8, 10, 14, 18, and 24 DAP for endosperm. Sample preparation, extraction and data evaluation were done as described by Weichert *et al.* (2010). Data was visualised with the VANTED software package (Junker *et al.*, 2006). To analyse micro-dissected grain vascular tissue, 9 nl material for eight biological replicates was collected at 4, 8, 10, 14, 18, and 24 DAP, extracted, and evaluated as described previously (Riewe *et al.*, 2012; Thiel *et al.*, 2012*b*).

## Results

### Growth parameters, starch and N content

**Fig. 1.**
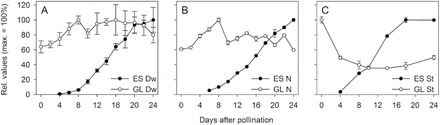
Changes of physiological parameters during endosperm (ES) and glume (GL) development. Relative changes (maximum amount = 100%) are shown for (A) dry weight (Dw), (B) total N, and (C) starch content (St) between 0 and 24 DAP. Data points represent three to five biological replicates ±SD.

Growth parameters were analysed for glumes between 0 and 24 DAP and for endosperm from 4 to 24 DAP. Glume dry weight increased steadily by 40% from 0 to 8 DAP followed by a transient decrease of 20% between 8 and 10 DAP. Thereafter, dry weight rose slightly, remained constant until 20 DAP and declined thereafter (Fig. 1A). Total N in glumes increased until 8 DAP, followed by a sharp decline at 8 DAP without further changes until 20 DAP and a slight decrease thereafter (Fig. 1B). Starch content in glumes was generally low compared to endosperm (Supplementary Table S1) and decreased by 65% between 0 and 10 DAP, before increasing by 15% from 18 to 24 DAP (Fig. 1C). Endosperm dry weight and starch content rose at ~8 DAP followed by linear accumulation during the main storage phase (10 to 20 DAP), levelling off afterwards (Fig. 1A, C). Total N increased linearly from 8 DAP until around 20 DAP (Fig. 1B).

The results show that from anthesis glumes accumulated dry weight and N until approximately 8 DAP, followed by a considerable decrease, coinciding with the start of starch and dry weight accumulation in the endosperm.

### Comparative gene expression analysis in glumes and endosperm

**Fig. 2.**
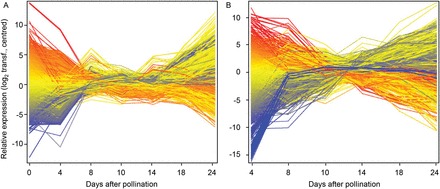
Expression profiles of 3999 and 8998 differentially expressed transcripts during development in (A) barley glumes and (B) endosperm, respectively. Data was derived from microarray experiments (Agilent 8×60K customized barley array); each time point represents three biological replicates. Raw expression values were log_2_ transformed, quantile normalized, and centred. Differential expression was detected by ANOVA (*P* < 0.005, *FC* > 3); single profiles were coloured according to their values at 0 DAP.

Comparative transcript analysis was performed in glumes and endosperm to analyse changes in central metabolic pathways, remobilization, and transport processes, as well as putative regulatory elements. Labelled cRNA from glume (0–24 DAP) and endosperm (4–24 DAP) fractions were hybridized to Agilent microarrays. In endosperm and glumes, 8998 and 3999 transcripts were identified as differentially expressed (significant differences between at least two stages; Supplementary Tables S2, S3). General profiles for both tissues are similar, revealing three distinct phases: (i) high differential expression between 0 and 8–10 DAP in glumes and endosperm; (ii) low differential expression between 8–10 and 14 DAP for glumes and endosperm; (iii) high differential expression after 14 DAP (Fig. 2A, B).

### Central carbohydrate and N metabolism

**Fig. 3.**
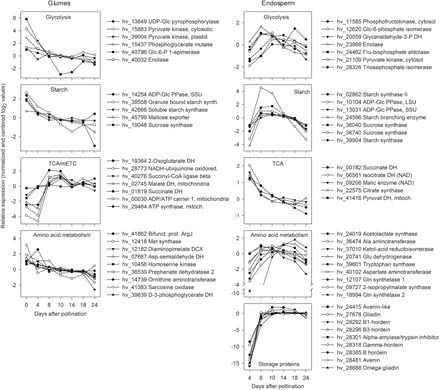
Comparison of transcript profiles between glumes (left panel) and endosperm (right panel) fractions. Relative expression values (means of three biological replicates, quantile normalized and baseline transformed) are shown for array contigs (hv_number) involved in central C and N metabolism, energy balance, and storage proteins (endosperm only). DCX, decarboxylase; DH, dehydrogenase; LSU, large subunit; PPase, pyrophosphorylase; SSU, small subunit.

In glumes, gene expression related to glycolysis (e.g. Glc-6-P epimerase, enolase, cytosolic/plastidic pyruvate kinase, and phosphoglycerate mutase) decreased steeply from 0 to 8 DAP and slightly thereafter. This was similar to the expression of the main starch metabolism genes, such as sucrose synthase, various starch synthases, and ADP-Glc pyrophosphorylase. By contrast, expression of seven genes related to glycolysis and seven associated with starch biosynthesis strongly increased in endosperm from 4 to 8 DAP, remained constantly high up to 14 DAP, and declined thereafter (Fig. 3).

In glumes between 4 and 8 DAP genes related to the tricarboxylic acid (TCA) cycle (2-OG dehydrogenase, succinyl CoA ligase, and malate dehydrogenase) and to the mitochondrial electron-transport chain (mETC) (succinate dehydrogenase, NAD:ubiquinone oxidoreductase, and ATP synthase), were steeply upregulated. In endosperm, expression of TCA cycle-related genes, such as citrate synthase, pyruvate dehydrogenase or NAD-isocitrate dehydrogenase, was highest at 4 DAP, decreased steadily until 10 DAP, and remained constant until 24 DAP (Fig. 3).

In glumes, nine genes associated with amino acid biosynthesis were most highly expressed at 0 and 4 DAP, followed by decreasing expression. Four are involved in the aspartate pathway towards lysine, methionine, and threonine biosynthesis; two others are involved in arginine biosynthesis. By contrast, endosperm expression of nine genes related to amino acid biosynthesis increased at 8 DAP and decreased after 18 DAP (Fig. 3). Endosperm expression of storage protein genes increased strongly from 4 to 8 DAP, and remained at a high level until 24 DAP (Fig. 3).

Transcript analysis revealed opposing trends for certain metabolic pathways such as glycolysis, and starch and amino acid synthesis, these being downregulated in glumes but upregulated in the endosperm during grain filling. TCA cycle and mETC-activities were strongly upregulated in glumes at 8 DAP, the beginning of grain filling.

### Carbohydrate and N transporters

**Fig. 4.**
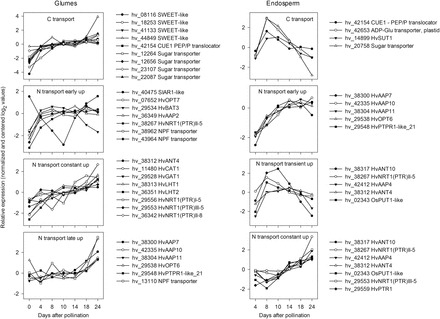
Expression patterns of putative C and N transporters in glumes (left panel) and endosperm (right panel). Relative expression values (see Fig. 3) are presented for putative carbohydrate, nitrate/peptide (NRT/PTR – NPF transporter, respectively), and amino acid (aa) transporters (subgroups: AAP, general aa permease; ANT, aromatic and neutral aa transporter; BAT, bidirectional aa transporter; CAT; cationic aa transporter; GAT, GABA transporter; LHT, lysine/histidine transporter; PUT, polyamine uptake transporter; SIAR, siliques are red – MtN21-like transporter).

Remobilization of reserves from glumes and accumulation in the endosperm depends on efficient transport from sink to source. In glumes, expression of several carbohydrate transporter genes increased at 8 DAP and then further until 24 DAP. This involved hexose/sugar transporters and members of the SWEET family, which potentially export sugars from *Arabidopsis* leaves (Chen *et al.*, 2012). In the endosperm, solute transporters related to storage product synthesis (e.g. HvSUT1, plastidic translocators for ADP-Glc and phosphoenol pyruvate) were upregulated at 8 DAP with decreasing expression levels towards 24 DAP (Fig. 4).

In glumes, more than 72% of putative amino acid transporters were at least transiently upregulated (Supplementary Table S4). Three major profiles were evident, early upregulated (between 0 and 8 DAP), constantly upregulated, and late upregulated (after 14 DAP) (Fig. 4).

*HvAAP2* was upregulated between 0 and 8 DAP and is homologous to *AtAAP2*, which is involved in xylem to phloem transfer and is important for sink N supply (Zhang *et al.*, 2010). *HvAAP7* and *HvAAP11* were upregulated from 14 and 18 DAP and are possibly involved in senescence-related remobilization. The second group contained members from other ATF subgroups like *HvLHT1*, specifically expressed in glumes and probably important for amino acid re-translocation (Kohl *et al.*, 2012), *HvCAT1*, *HvANT4*, and *HvGAT1*. Barley homologues of AtBAT1 (Dündar and Bush, 2009) and AtSIAR1 (Ladwig *et al.*, 2012) showed opposing expression profiles. *HvBAT3* was upregulated between 0 and 14 DAP followed by downregulation, while *HvSIAR1-like* was downregulated between 0 and 10 DAP followed by strong upregulation.

Bias towards upregulation is less pronounced for putative nitrate/peptide transporters, where 13 from 30 candidates showed decreasing expression (Supplementary Table S4). Within upregulated candidates, three major profiles present in the ATF transporters could be observed (Fig. 4). Endosperm-expressed N transporters probably facilitate N import or distribution. 80% and >73% of ATF and NPF transporters, respectively, were upregulated during development (Supplementary Table S5). Major patterns showed upregulation until 14 DAP, transient upregulation at 8 or 10 DAP, and constant upregulation after 8 DAP (Fig. 4).

Among transiently upregulated transcripts, *HvAAP3* is closely related to *AtAAP8* and *AtAAP1*, importing amino acids into seeds (Schmidt *et al.*, 2007; Sanders *et al.*, 2009). Expression of a *BAT-like* transcript was also steadily increasing during development, and is possibly involved in phloem unloading (Dündar, 2009), while increasing expression of *OsPUT1-like* after 8 DAP indicates polyamine import into grains (Mulangi *et al.*, 2012).

### Transcriptional transitions in glume metabolism during grain filling

**Fig. 5.**
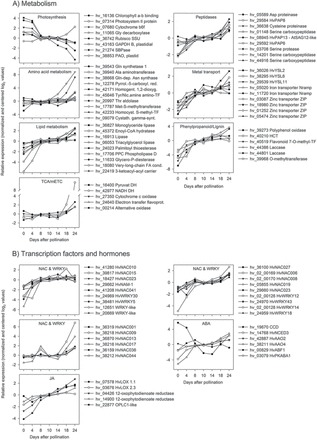
Expression patterns of components regulating transition in glumes from sink to source tissue. Relative expression values (see Fig. 3) are shown for selected transcripts: (A) metabolism; (B) transcription factors and hormones. ABF, ABA-responsive element binding factor; AAO, aldehyde oxidase; CCD, carotenoid cleavage dioxygenase; HCT, hydroxycinnamoyl-CoA shikimate/quinate hydroxycinnamoyl transferase; LOX, lipoxygenase; NCED, 9-*cis*-epoxycarotenoid dioxygenase; PAO, pheophorbide a oxygenase; PAP, papain-like cysteine peptidase; PC, phosphatidylcholine; PKABA, ABA-inducible protein kinase; SBPase, sedoheptulose-1,7-bisphosphatase; TF, transferase; YSL, yellow-stripe-like transporter.

Photosynthesis-associated transcripts in glumes were highly expressed at 0–4 DAP, with decreasing levels after 8 DAP (Fig. 5A). Chlorophyll is degraded during leaf senescence by a pathway involving pheophorbide a oxygenase (PAO) (Hörtensteiner, 2006). In glumes, *HvPAO* was upregulated from 14 DAP onwards (Fig. 5A), indicating chlorophyll degradation only at late seed filling. Protein degradation is a prerequisite for N remobilization, and several proteases were transcriptionally upregulated in glumes at two distinct phases, between 0 and 8 DAP and from 14 DAP onwards. This involved several serine-, aspartyl-, and cysteine-like proteinases including a homologue to *Arabidopsis senescence-associated gene-12* (*SAG12*), which is specifically activated by developmentally controlled senescence but is not stress- or hormone-controlled (Noh and Amasino, 1999).

Whereas several amino acid biosynthesis genes were downregulated (Fig. 3), others involved in different parts of amino acid metabolism (e.g. homogentisate 1,2-dioxygenase and pyrroline-5-carboxylate reductase) were upregulated in glumes and may participate in aromatic amino acid and proline degradation. Alanine aminotransferase and glutamine synthetase-1 (GS1) could be involved in glutamine biosynthesis for export (Thiel *et al.*, 2009). Thus, expression patterns indicate interconversion and/or degradation of certain amino acid species in glumes during later grain filling. Transcripts of glutamine-dependent asparagine synthase (Gln-ASN) increased by 150-fold between 14 and 24 DAP, indicating an important role for remobilization (Fig. 5).

Potential metal transporters, like members of natural resistance-associated macrophage proteins (NRAMP), zinc transporters, yellow stripe-like (YSL), and oligopeptide transporters (OPT) were transcriptionally upregulated at 8 DAP and/or at 24 DAP. Specific genes of lipid biosynthesis/degradation were differently expressed during glume development. Two members degrading phospholipids, phosphatidylcholine phospholipase D, and glycero-P-diesterase were strongly upregulated between 0 and 8/10 DAP. Candidates involved in degradation of mono- and triacylglycerides, like enoyl-CoA hydratase, palmitoyl protein thioesterase, and mono- and triacylglycerol lipase were upregulated after 14 DAP. Enzymes synthesizing long-chain fatty acids, acyl-activating enzyme, very-long-chain fatty acid-condensing enzyme, and 3-ketoacyl-acyl carrier protein were most highly expressed at 18 and 24 DAP.

Genes involved in the phenylpropanoid/lignin pathway were upregulated in glumes from 8 to 24 DAP, including polyphenol oxidase, flavonoid 7-O-methyltransferase, and hydroxycinnamoyl-CoA shikimate/quinate hydroxycinnamoyl transferase. Two genes encoding laccases were constantly upregulated during development. Laccases catalyse lignin polymerization from the precursors coniferyl- and sinapyl-alcohol, and respective knockouts in *Arabidopsis* drastically reduce the lignin content (Zhao *et al.*, 2013), indicating an important role for lignification and secondary cell wall thickening in mechanical support and water transport (Zhao *et al.*, 2013).

Whereas mitochondrial activity was transcriptionally activated at 8 DAP (Fig. 3), specific genes encoding TCA-cycle and mETC-enzymes, such as pyruvate and NADH dehydrogenases, electrontransfer flavoprotein, and alternative oxidase, were upregulated only after 14 DAP.

Results from transcript profiling suggest metabolic transitions in glumes from sink to source in accordance to grain filling, namely downregulated photosynthesis and upregulated proteolysis, lipid and phenylpropanoid metabolism, and mitochondrial activities and metal transport.

### Transcriptional and hormonal control of remobilization in glumes

NAC and WRKY transcription factors are frequently involved in senescence signalling (Zentgraf *et al.*, 2010; Breeze *et al.*, 2011; Christiansen and Gregersen, 2014). Most NAC transcription factors were upregulated, showing three major profiles: (i) upregulation until 8 DAP, with constant levels afterwards, including *HvNAM-1*, homologous to *TtNAM-1*, a regulator of senescence and remobilization in wheat flag leaves (Uauy *et al.*, 2006; Distelfeld *et al.*, 2008); (ii) bi-phasic upregulation, with increasing transcript abundances between 0 and 8 DAP and after 14 DAP, including *HvNAC006* and *HvNAC008*, homologous to *AtORE1* and *AtATAF1*, controlling leaf senescence in *Arabidopsis* (Kim *et al.*, 2009; Kleinow *et al.*, 2009; Balazadeh *et al.*, 2010); (iii) upregulation after 14 DAP obviously associated with developmental senescence (Fig. 5B). The latter group includes *HvNAC009* and *HvNAC013*, upregulated in old ears and grains and old leaves, and inducible by methyl-jasmonate (Christiansen *et al.*, 2011).

Nineteen out of 22 WRKY transcription factors showed profiles according to (i) and (ii). The latter includes *HvWRKY12*, which is probably involved in age-dependent senescence rather than N remobilization (Hollmann *et al.*, 2014).

Hormones affect leaf senescence differently; senescence is delayed by cytokinins (CKs) and gibberellic acids (GAs), and accelerated by abscisic acid (ABA) and jasmonates (JA) (Jibran *et al.*, 2013). Transcripts for carotenoid cleavage dioxygenase, involved in early ABA biosynthesis, decreased until 10 DAP and increased after 14 DAP. Genes involved in late ABA biosynthesis, 9-*cis*-epoxycarotenoid dioxygenase (*HvNCED3*) and aldehyde oxidase (*HvAAO*), were upregulated bi-phasically between 0 and 8 DAP and after 14 DAP. ABA-inducible protein kinase (*HvPKABA1*) and ABA-responsive element binding factor 1 (*HvABF1*) were similarly upregulated bi-phasically and are possibly involved in mediating GA/ABA responses (Yamauchi *et al.*, 2002; Schoonheim *et al.*, 2009).

JA biosynthesis depends on the subsequent action of lipoxygenase (LOX), allene oxide synthase and cyclase, 12-oxophytodienoate reductase (OPR), followed by beta oxidation (Lyons *et al.*, 2013). In glumes, continuously upregulated genes encode specific LOX isoforms and enzymes involved in late steps of JA biosynthesis (OPRs) and a homologue to AtOPLC1, involved in beta oxidation (Koo *et al.*, 2006).

### Metabolite contents in developing glumes and endosperm

**Fig. 6.**
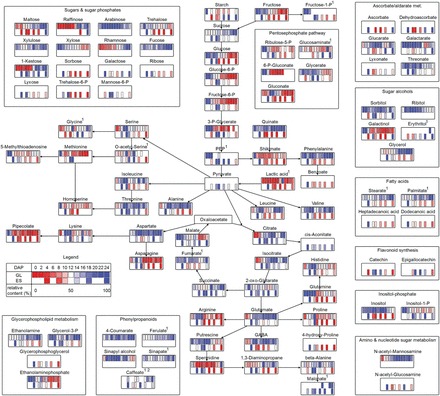
Changes in metabolite levels in barley glumes and endosperm during development. Samples were taken in 2-day steps between 0 and 24 DAP (glumes) and at 4, 8, 10, 14, 18, and 24 DAP (endosperm). Levels of proteogenic amino acids and GABA were measured using UPLC with two (Arg, Gly) and three biological replicates, respectively. All other metabolites were measured by GC-MS with six biological replicates per time point. Data were corrected by internal standard and fresh weight, subsequently maximum normalized and colour coded for each metabolite (maximum amount = 100%, values >90% coloured in dark blue, values <10% in dark red), as shown by the insert depicting a constant increase from 0 to 100% between 0 and 24 DAP. Metabolites without significant changes in one of the tissues are marked (1) for glumes and (2) for endosperm.

Metabolite levels were measured in glumes and endosperm (Fig. 6, Supplementary Table S6), while free amino acids were analysed by UPLC (Supplementary Table S7). Levels of sucrose, maltose, raffinose, xylulose, rhamnose, and fucose increased whereas xylose, arabinose, and trehalose decreased in glumes with progressing development. Several sugars are involved in cell wall biosynthesis, indicating alterations in cell wall dynamics in glumes. In endosperm, hexoses were highest during early development whereas sucrose peaked at 8 DAP (Weschke *et al.*, 2000).

In glumes, almost all metabolites within the glycolytic, pentose phosphate, and shikimate pathways, like hexoses and their phosphates, 6-phospho-gluconate and 3-phospho-glycerate, decreased whereas endosperm levels were highest at 8 and 10 DAP. Inositol and inositol-1-phosphate and sugar alcohols, except glycerol, decreased during glume development.

TCA intermediates, except 2-oxoglutarate and malate, increased in glumes, while the highest endosperm levels occurred at early and mid-development. Phenylpropanoid intermediates 4-coumarate, caffeate, ferulate, and sinapate also rose during glume development. Metabolites related to glycerophospholipid metabolism (ethanolamine, glycerol-3-P) increased in glumes whereas ethanolaminephosphate decreased. In the endosperm all these metabolites decreased over time. Fatty acids (stearate, palmitate) rose in glumes while endosperm levels declined. GABA, putrescine, 1, 3-diaminopropane, and β-alanine increased in glumes from 0 DAP to 18 DAP, while spermidine decreased. In the endosperm all these metabolites decreased (Fig. 6).

### Free amino acid concentrations in glumes, endosperm, and vascular tissue

**Fig. 7.**
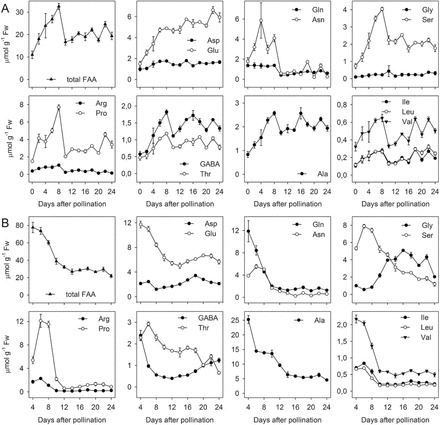
Concentrations of free amino acids in (A) barley glumes and (B) endosperm during development. Samples were measured via UPLC in 2- day steps from 0 DAP (glumes) and 4 DAP (endosperm), respectively, before data were corrected by internal standard and fresh weight. Each data point represents two (Arg, Gly) or three biological replicates ±SD.

In glumes between pollination and 8 DAP, the summarized concentrations of all free amino acids increased from 11 to 32 µmol g^–1^ fresh weight, followed by a drastic decrease between 8 and 10 DAP (~50%; Fig. 7A; Supplementary Table S7). Thereafter, levels fluctuated at around 20 µmol g^–1^. Most amino acids behaved similarly, showing distinct declines between 8 and 10 DAP, except Asp and Glu. Asp was generally low without larger changes, whereas Glu increased throughout. Ser was always higher by 10- to 15-fold compared to Gly. Asn was 2 to 3-fold higher than Gln between 2 and 10 DAP but not different later on.

In the endosperm, free amino acid levels decreased from 80 µmol g^–1^ fresh weight at 4 DAP to 25 µmol g^–1^ at 14 DAP, showing the largest decline between 8 and 10 DAP (Fig. 7B). Between 16 and 22 DAP levels remained constant, before decreasing at 24 DAP. While Ala, Gln, Glu, and Val followed this pattern, Arg, Asn, Pro, Ser, and Thr accumulated between 4 and 6 DAP, before their levels declined.

Concentrations of Gln and Pro most strongly declined (by ~80%) between 4 and 12 DAP. In contrast to Ser, Gly increased from 8 to 12 DAP, marking a switch from high to low Ser:Gly ratios during grain filling. Asp largely did not change. The results indicate a sudden decrease of most free amino acids (especially of Pro and Asn) in glumes at 8 DAP. This coincides with extensive use for storage protein synthesis in the endosperm.

**Fig. 8.**
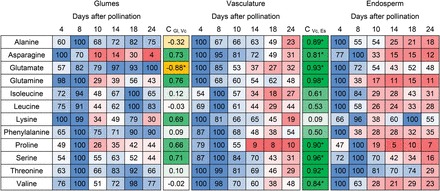
Relative concentrations of free amino acids in barley glumes (Gl), grain vasculature (Vc), and endosperm (Es). Measurements were taken between 4 and 24 DAP using UPLC for glumes and endosperm, with three biological replicates, and using GC-MS for grain vasculature, with eight biological replicates. Concentrations were normalized and colour coded from dark red (low) to dark blue (high) values (see Fig. 6), before Pearson correlation (C) was determined for Vc and Gl and Vc and Es, respectively. Correlations are shown in orange (negative) and green (positive); statistically significant correlations (*P* < 0.05) are marked with an asterisk.

As expected, high endosperm demand for amino acids due to storage activity is transmitted to the glumes, initiating remobilization. To analyse possible metabolic signals, GC-MS-based metabolic profiling was performed on micro-dissected regions comprising the main vascular bundles of grains between 4 and 24 DAP (Supplementary Figure S1). Thirty-two unambiguous metabolites were detected (Supplementary Table S8). Temporal profiles of 12 amino acids were compared between glumes, endosperm, and vascular regions (Fig. 8). Amino acid profiles were highly correlated (Pearson correlation) between vasculature and endosperm with C_vc, en_ between 0.98 and 0.81 for Gln, Ser, Glu, Thr, Pro, Ala, Val, and Asn. Less positive (C_vc, en_ <0.76) or even negative correlation (Ala, Glu) occurred between vasculature and glumes. This indicates that endosperm amino acid demand is propagated via the vasculature. In beech, increased Gln and Asp levels in the phloem lead to reduced root NO_3_^–^ uptake (Geßler *et al.*, 1998) and amino acid feeding negatively regulates expression of high-affinity NO_3_^–^-uptake transporters in barley roots (Vidmar *et al.*, 2000). In a reciprocal manner, depletion of certain amino acids such as Gln (C_vc, en_ = 0.98) within the vasculature could signal and communicate endosperm demand to vegetative organs.

## Discussion

Glumes are the vegetative organs closest to grains and are important for converting and translocating assimilates to them (Waters *et al.*, 1980; Simpson *et al.*, 1983; Lopes *et al.*, 2006). Phase changes during endosperm development are accompanied by large variations of sink strength that greatly affect metabolism in and assimilate fluxes from glumes. Parallel transcript and metabolite profiling in glumes and endosperm during grain filling showed that glume metabolism was adjusted to the changing demands of the grains, reflected by specific signatures of metabolite and transcript abundances. Obviously, grain filling and filial sink strength coordinate phase changes in glumes via metabolic, hormonal, and transcriptional control.

### Glumes are photosynthetically active sinks during the pre-storage phase

Transcript profiling in glumes at 0 and 4 DAP revealed high activity of photosynthesis, glycolysis, and starch and amino acid biosynthesis, but low mitochondrial activity and transport of sugars, peptides and metals. Accordingly, levels of hexoses, their phosphates, glycolytic intermediates, and starch were highest. By contrast, endosperm gene expression was low for central pathways glycolysis, and starch and amino acid biosynthesis, but high for mitochondrial activity before 8 DAP (Figs 3–5). Thus, during the pre-storage phase, glumes are photosynthetically active organs with a high level of biosynthesis, while the endosperm is a sink with high respiratory and mitochondrial activities.

During the pre-storage phase, glumes accumulated dry matter, total N, and free amino acids, with concentrations increasing nearly 3-fold between 0 and 8 DAP (Fig. 7). Likewise, α-amino N content in wheat glumes increases significantly until 5 DAP (Waters *et al.*, 1980). This demonstrates that during the pre-storage phase glumes generate early and intermediary sinks before high endosperm sink strength is established.

### Glumes and endosperm display opposed metabolic shifts at the beginning of grain filling

At 8–10 DAP metabolic shifts occurred in glumes, indicated by decreasing dry matter, starch, total N, and most amino acids. At the transcript level this was reflected by downregulated photosynthesis, starch and amino acid biosynthesis, and glycolysis, but upregulated TCA cycle, mETC activities, and transport (Figs 3–5). Endosperm dry weight and starch increased linearly after 8 DAP together with storage-associated/sink-strength-related gene expression such as sucrose and amino acid transporters *HvSUT1*, *HvAAP3*, sucrose synthase, ADP-Glc pyrophosphorylase, and hordeins (Figs 3 and 4). Accordingly, levels of sucrose, glycolytic intermediates, and amino acids were highest at 8–10 DAP in endosperm but decreased in glumes (Fig. 6). To conclude, metabolic shifts implicate opposing trends for central pathways glycolysis, and starch and amino acid biosynthesis, namely downregulation in glumes but upregulation in endosperm at early grain filling.

Glumes undergo transition into remobilizing and exporting organs coinciding with the beginning of storage activity in the endosperm. Phase changes in glumes may be initiated by emerging endosperm sink strength. This is supported by the fact that removal of sink organs generally prevents remobilization and delays senescence. Amino acid concentrations (especially Gln) decrease upon grain filling in wheat flag leaves, but increase in response to ear excision (Peeters and Van Laere, 1994), indicating that grain sink strength induces this drain. Senescence is also delayed by diverse crop manipulations such as inhibiting kernel set in maize and depodding (Miceli *et al.*, 1995; Borrás *et al.*, 2003). Instead, senescence in spinach plants is induced by exhaustive reallocation of nutrients from leaves to flowers (Sklensky and Davies, 2011).

### Metabolic transition of glumes occurs in two phases

Gene expression in glumes suggested metabolic transitions at two phases. The first, at around 8 DAP, is consistent with the onset of endosperm storage activity and is probably a consequence of increasing endosperm sink activity. Amino acid profiles in the grain vasculature were highly correlated to those of the endosperm, but differed from glumes (Fig. 8). Although the total amount of free amino acids in the endosperm increased until 18 DAP (data not shown), concentrations of most members decreased during early grain filling. Such depletion might be transmitted via the vasculature and specific amino acids could function as metabolic signals communicating endosperm demand to vegetative organs. Chlorophyll breakdown, regarded as a senescence marker (Hörtensteiner, 2006), did not occur at 8 DAP since pheophorbide a oxygenase (PAO) was transcriptionally activated after 14 DAP. Senescence-associated genes, like cysteine proteinase, homologous to *Arabidopsis* SAG12 (Guo and Gan, 2005) and an AtSWEET15 homologue (AtSAG29), a potential sugar exporter and senescence-related protein that accelerates senescence when overexpressed in *Arabidopsis* (Seo *et al.*, 2011), were also not upregulated before 14 DAP. To conclude, developmental senescence in glumes was not initiated before 18 DAP (Fig. 5).

The second phase comprised developmental ageing and senescence denoting later grain filling. At transcript levels it was characterized by a further decrease of photosynthesis, glycolysis, and starch biosynthesis, whereas chlorophyll, lipid, and amino acid degradation increased together with proteolysis, mETC activity, and transport processes. Several genes involved in the final steps of amino acid biosynthesis were steadily downregulated, while others were upregulated, especially at later stages, such as homogentisate 1,2-dioxygenase and pyrroline-5-carboxylate reductase, which are probably engaged in Tyr, Phe, and Pro degradation.

To conclude, upon the first metabolic transition (8 DAP), glumes are converted into remobilizing and transporting organs for assimilates, providing for grain filling. The second transition (18 DAP) assigns developmental ageing and senescence. The period between is the main storage phase. It is obviously important that glumes remain fully functional at this stage. Sequential arrangements reflect the cascades of sink-induced remobilization at 8 DAP and developmental ageing after 14 DAP.

### Regulation of glume metabolic transitions

NAC and WRKY transcription factors are frequently involved in signalling senescence (Uauy *et al.*, 2006; Zentgraf *et al.*, 2010; Breeze *et al.*, 2011; Christiansen and Gregersen, 2014). In glumes, three major patterns of upregulation are evident.

*HvNAM-1* was upregulated at 8/10 DAP and influences grain protein content (Jamar *et al.*, 2010), while the wheat homologue, TtNAM-1, effects senescence and remobilization in flag leaves (Uauy *et al.*, 2006; Distelfeld *et al.*, 2008). Thus, *HvNAM-1* could be involved in adjusting glume metabolism in response to grain filling. *HvNAC001*, *HvNAC013*, *HvNAC036*, and *HvNAC044* were upregulated after 14 DAP and are induced during leaf senescence (Christiansen *et al.*, 2011). In glumes, these four NACs could initiate chloroplast degeneration, executed by co-induced HvPAO and SAG12-like proteinase. Twenty-one NACs and WRKYs were bi-phasically upregulated between 0 and 8 DAP and after 14 DAP. *HvNAC006* and *HvNAC008* are homologous to *AtORE1* and *AtATAF1*, controlling *Arabidopsis* leaf senescence (Kim *et al.*, 2009; Balazadeh *et al.*, 2010), and initiating early senescence upon overexpression (Kleinow *et al.*, 2009), respectively.

Biosynthesis and signalling of ABA are upregulated during senescence (van der Graaff *et al.*, 2006). *HvNCED3* was continuously upregulated and *HvAAO4* expression increased from 0 to 8 DAP and after 14 DAP, indicating that late steps of ABA biosynthesis are upregulated in glumes. Accordingly, ABA signalling, *HvPKABA1*, and *HvABF1* were bi-phasically upregulated, supporting ABA functions in glume phase transition. JA generally accelerates leaf senescence (Ueda *et al.*, 1981), although mechanisms are still unclear. MeJA induces senescence-associated transcripts *AtSAG12* (Xiao *et al.*, 2004), *AtCLH1/CORI1*, and *AtERD1/SAG15* (Jung *et al.*, 2007). Enzymes involved in JA biosynthesis, OPRs, lipoxygenases, and the AtOPLC1-homologue, involved in JA-related β-oxidation (Koo *et al.*, 2006), were continuously upregulated in glumes. In contrast to ABA, JA-related transcripts were not bi-phasically upregulated, indicating effects only on age-dependent senescence.

### Glume transition from sink to source is accompanied by changed expression of N transporters

Switching from import to remobilization/export requires transport/re-translocation of N. *HvAAP2*, upregulated at 8/10 DAP, is homologous to *AtAAP2*, and involved in *Arabidopsis* xylem-to-phloem transfer and sink N supply (Zhang *et al.*, 2010). NPF-members hv_38962 (array contig) and hv_38267 were also upregulated at 8/10 DAP and are similar to tonoplast-localized AtPTR2/AtNPF8.3, functioning in flowering and seed development (Song *et al.*, 1997), and to plasma membrane-localized AtPTR5/AtNPF8.2, important for peptide transport in seeds (Komarova *et al.*, 2008). These transporters could potentially establish sink strength and intermediate storage in glumes (Fig. 4).

Three putative LHTs were upregulated and potentially involved in amino acid remobilization within glumes (Fig. 4). Their homologues, AtLHT1 and AtLHT2, import amino acids into *Arabidopsis* leaf mesophyll and tapetum cells (Lee and Tegeder, 2004; Hirner *et al.*, 2006). Constantly upregulated NPF transporters hv_29556 and hv_36342 (Fig. 4) could import nitrate into glumes similarly to *Arabidopsis* homologues AtNRT1.4/NPF6.2, which accumulates nitrate in leaf petioles (Chiu *et al.*, 2004), and AtNRT1.8/NPF7.2, involved in xylem unloading (Li *et al.*, 2010).

Four AAPs were upregulated in glumes after 14 DAP (Fig. 4). HvAAP11 is related to AtAAP5, involved in phloem loading (Fischer *et al.*, 1995). Thus, HvAAP11 is probably exporting amino acids during senescence-associated proteolysis in glumes. HvAAP10 is homologous to AtAAP6, involved in xylem-to-phloem transfer (Okumoto *et al.*, 2002). HvAAP10 probably relocates amino acids to vascular tissue and developing grains.

NPF-like transporters hv_29548 and hv_13110 were upregulated only after 14 DAP. Corresponding *Arabidopsis* homologues AtNRT1.5/NPF7.3 and AtNRT1.11/NPF1.2 are involved in xylem loading (Lin *et al.*, 2008) and xylem-to-phloem transfer of nitrate (Hsu and Tsay, 2013), respectively. These transporters could translocate nitrate in glumes.

Putative amino acid exporters *HvBAT3* and *HvSIAR1-like* were opposingly expressed (Fig. 4). *HvBAT3*, downregulated after 10 DAP, is homologous to *AtBAT1*, putatively involved in sink phloem unloading (Dündar and Bush, 2009). *HvSIAR1-like*, upregulated after 14 DAP, is homologous to *AtSIAR1*, and involved in amino acid remobilization and homoeostasis of *Arabidopsis* leaves (Ladwig *et al.*, 2012). Switching activities of *HvBAT3* and *HvSIAR1-like* could reflect the transition of glumes from sink to source.

*HvOPT6*, with unique expression among oligopeptide transporters, was downregulated at 4 DAP and upregulated after 14 DAP, and is homologous to glutathione transporter *OsGT1* (Zhang *et al.*, 2004), suggesting combined N and sulphur remobilization.

### Glume-specific remobilization of assimilates and resources

**Fig. 9.**
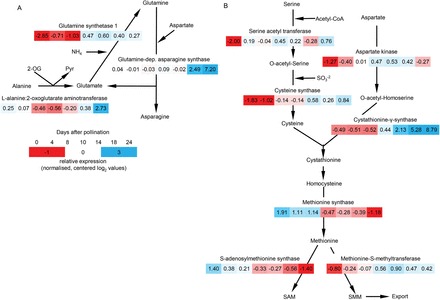
Possible glume-specific mechanisms for remobilization of N and S. This shows conversion of alanine by Ala:OG-AT to glutamate and further to glutamine by GS1 using amino groups from protein/amino acid degradation (A) and conversion of amino N from serine, aspartate, and sulphur into the phloem-mobile SMM (B). Normalized, relative expression values are presented and colour coded from low (dark red) to high (dark blue) expression.

Gene expression and metabolite profiles indicate glume-specific mechanisms of assimilate conversion and translocation towards grains. Induced L-alanine:2-oxoglutarate aminotransferase (Ala:OG-AT) and GS1 together may convert Ala by Ala:OG-AT to Glu and further to Gln by GS1 using amino groups from protein/amino acid degradation. Gln could then either be exported or converted to Asn by Gln-ASN, one of the most upregulated genes at 24 DAP (150-fold). This would mobilize N as Asn at the expense of Ala and amino N. Gln-ASN is important for remobilizing N during senescence of *Medicago truncatula* leaves (De Michele *et al.*, 2009). In rice and tobacco, Gln-ASN is located in vascular tissues (Gaufichon *et al.*, 2010). Assuming such a location in glumes suggests that Asn is preferentially synthesized in vascular tissue for export (Fig. 9A). Accordingly, levels of Ala and Glu remain high in glumes during grain filling, whereas Asn decreases 5-fold with similar profiles in glumes, vasculature, and endosperm.

Cystathionine-γ-synthase (CGS), involved in Met biosynthesis (Hacham *et al.*, 2013), was upregulated at 24 DAP in glumes (100-fold) together with serine acetyl transferase (SAT) and cysteine synthase (OAS1), involved in Cys biosynthesis from Ser. Upregulated Cys and Met biosynthesis contributes to possible conversion of amino N from Ser, Asp, and sulphur to phloem-mobile *S*-methyl-methionine (SMM), (Bourgis *et al.*, 1999). The pathway (Fig. 9B), involves additional enzymes, like aspartate kinase and methionine-*S*-methyltransferase, which were also upregulated in glumes. Similar mechanisms are suggested for synthesis and transport of SMM within barley nucellar projections to translocate reduced sulphur from senescing tissue into endosperm (Thiel *et al.*, 2009).

Differential transcription indicated degradation of phospholipids by phosphatidylcholine phospholipase D and glycero-*P*-diesterase, in glumes already at 8 DAP, together with biosynthesis of long-chain fatty acids by acyl-activating enzyme and very-long-chain fatty acid-condensing enzyme. However, degradation of mono- and triacylglycerides (TCGs) did not occur before 18 DAP, indicated by upregulated mono- and triacylglycerol lipases. These processes are probably involved in mobilizing C from membrane lipids into phloem-mobile sucrose (Kaup *et al.*, 2002; Troncoso-Ponce *et al.*, 2013). To conclude, in glumes, phospholipids from chloroplasts are degraded early, accompanied by *de novo* biosynthesis of TCGs. Accordingly, levels of stearate and palmitate increased in glumes (Fig. 6). During developmental senescence TCGs are degraded and C is converted to phloem-mobile sucrose or respired by mETC. Alternatively, acetyl-CoA could be converted into amino acids and to SMM for export (Fig. 9B).

In glumes, mitochondrial metabolism was upregulated at early grain filling involving TCA cycle activity, mETC, and ATP synthesis and transport, consistent with increased levels of citrate, isocitrate, and succinate. As expected, most ATP generated in mitochondria is needed to energize transport of sugars, amino acids, and peptides. Accordingly, respective transporters were co-induced (Fig. 4). Similarly, in senescing *Arabidopsis* leaves, mitochondrial respiration has to supply ATP and C skeletons to redistribute N (Hörtensteiner and Feller, 2002). Several mitochondrial genes were only upregulated later on, involving respiration and mETC (cytochrome c oxidase, electron transfer flavoprotein, NADH dehydrogenase) and energy dissipation (alternative oxidase). These genes are probably involved in developmental senescence and amino acid degradation, also upregulated at the transcript level at 24 DAP. Mitochondrial alternative oxidase could balance senescence-related stress responses from excess degradation of sugars and/or amino acids as shown in legume embryos with perturbed metabolism (Gregersen and Holm, 2007; Weigelt *et al.*, 2008; Weigelt *et al.*, 2009; Araújo *et al.*, 2014).

Our results show that development of barley glumes after anthesis is separated into three phases associated with grain development, and these mark the transition from sink to source tissue. Until 8 DAP, glumes are growing and photosynthetically active tissues accumulating dry weight, total N, and free amino acids. Furthermore, decreasing levels of starch and glycolytic metabolites as well as the corresponding transcripts are observed. Between 8 and 10 DAP, coinciding with the beginning of storage protein synthesis in grains, total N and free amino acids decrease significantly, which probably represents relocation of nutrients to meet the demands of developing grains. Concentrations of free amino acids in endosperm and grain vasculature decrease at this stage, which could signal increasing N demand to glumes and trigger remobilization. Accordingly, expression of specific transporters in glumes is upregulated together with enzymes from the TCA cycle and mETC providing energy for transport. After 18 DAP, glumes undergo developmental ageing and senescence, involving chlorophyll degradation by PAO, specific proteases, and N transporters. Transition between these phases is probably governed by transcription factors from the NAC and WRKY families and influenced by ABA.

## Supplementary material

Supplementary data can be found at *JXB* online.

Supplementary Table 1. Profiles for dry weight, total N, and starch.

Supplementary Table 2. Differentially expressed transcripts in glumes.

Supplementary Table 3. Differentially expressed transcripts in endosperm.

Supplementary Table 4. Differentially expressed N transporters in glumes.

Supplementary Table 5. Differentially expressed N transporters in endosperm.

Supplementary Table 6. Metabolite profiles (GC-MS) in glumes and endosperm.

Supplementary Table 7. Amino acid profiles (UPLC) in glumes and endosperm.

Supplementary Table 8. Metabolite profiles (GC-MS) in grain vasculature.

Supplementary Figure 1. Light microscopic images showing micro-dissection of vascular tissue.

## Funding

This work was supported by the Deutsche Forschungsgemeinschaft (DFG) in the frame of Research Group 948: Nitrogen uptake, metabolism, and remobilization in leaves during plant senescence (Grant Number: WE 1641/13-2)

## Supplementary Material

Supplementary Data
