# Supplementary material for: Metabolic and transcriptional transitions in barley glumes reveal a role as transitory resource buffers during endosperm filling
Source: J Exp Bot. 2015 Jan 22;66(5):1397–411. doi: 10.1093/jxb/eru492 (PMC4339599; doi:10.1093/jxb/eru492)
Supplement: Supplementary Data [file supp_66_5_1397__index.html]

Metabolic and transcriptional transitions in barley glumes reveal a role as transitory resource buffers during endosperm filling — Metabolic and transcriptional transitions in barley glumes reveal a role as transitory resource buffers during endosperm filling — Supplementary Data 

# Metabolic and transcriptional transitions in barley glumes reveal a role as transitory resource buffers during endosperm filling

## Supplementary Data

Data files

**Files in this Data Supplement:**

- Supplementary Data - Supplementary Data
- Supplementary Data - Supplementary Data
- Supplementary Data - Supplementary Data
- Supplementary Data - Supplementary Data
- Supplementary Data - Supplementary Data
- Supplementary Data - Supplementary Data
- Supplementary Data - Supplementary Data
- Supplementary Data - Supplementary Data
- Supplementary Data - Supplementary Data
