# Supplementary material for: Metabolic and transcriptional transitions in barley glumes reveal a role as transitory resource buffers during endosperm filling
Source: J Exp Bot. 2015 Jan 22;66(5):1397–411. doi: 10.1093/jxb/eru492 (PMC4339599; doi:10.1093/jxb/eru492)
Supplement: Supplementary Data [file supp_eru492_jexbot132944_file009.pdf]

Metabolic and transcriptional transitions in barley glumes reveal a role as transitory buffer of resources during endosperm filling

S. Kohl, J. Hollmann, A. Erban, J. Kopka, D. Riewe, W. Weschke, H. Weber

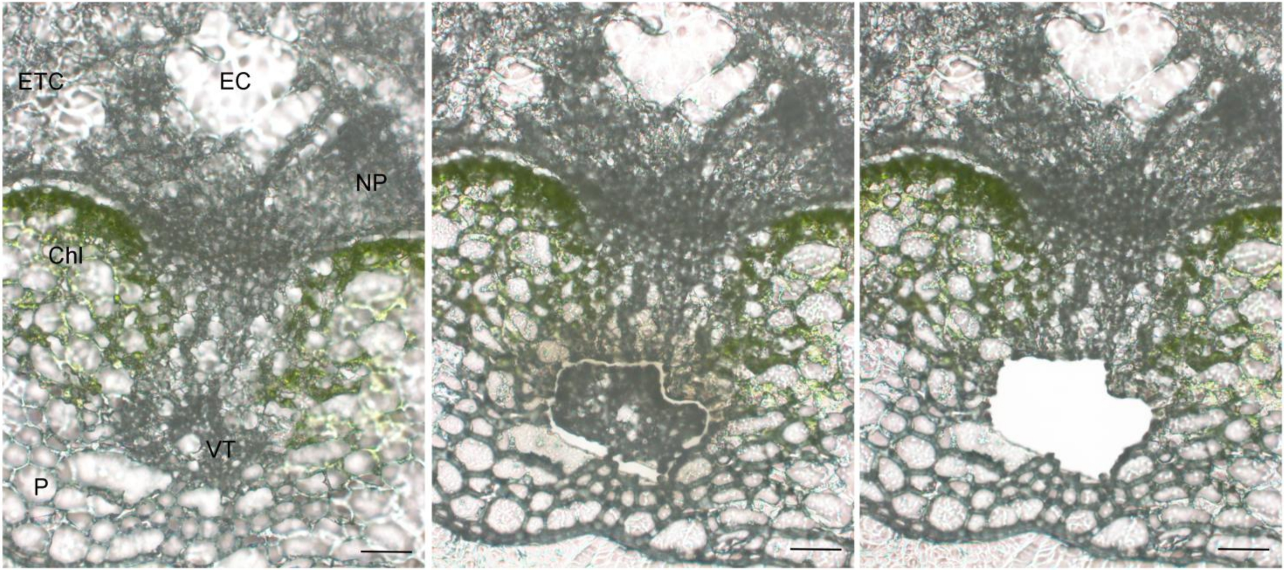

Figure S1: Cross-section through a developing barley grain at 8 DAP. Light microscopic pictures visualising the process of micro dissection: intact grain (left), grain with dissected vasculature (middle) and grain with dissected and removed vasculature (right). Scale bar represents 100µm; pictures were taken with Olympus IX81 microscope at 20x magnification. Chl, chlorenchyma; EC, endospermal cavern; ETC, endosperm transfer cells; NP, nucellar projection; P, pericarp; VT, vascular tissue.
